# Funding of Hispanic/Latino Health-Related Research by the National Institutes of Health: An Analysis of the Portfolio of Research Program Grants on Six Health Topic Areas

**DOI:** 10.3389/fpubh.2020.00330

**Published:** 2020-08-28

**Authors:** M. Larissa Avilés-Santa, Laura Hsu, Tram Kim Lam, S. Sonia Arteaga, Ligia Artiles, Sean Coady, Lawton S. Cooper, Jennifer Curry, Patrice Desvigne-Nickens, Holly L. Nicastro, Adelaida Rosario

**Affiliations:** ^1^Clinical and Health Services Research, National Institute on Minority Health and Health Disparities, Bethesda, MD, United States; ^2^Division of Extramural Research Activities, National Heart, Lung, and Blood Institute, Bethesda, MD, United States; ^3^Division of Cancer Control and Population Sciences, National Cancer Institute, Bethesda, MD, United States; ^4^Division of Cardiovascular Sciences, National Heart, Lung, and Blood Institute, Bethesda, MD, United States; ^5^Division of Scientific Programs, National Institutes on Minority Health and Health Disparities, Bethesda, MD, United States; ^6^Center for Translation Research and Implementation Science, National Heart, Lung, and Blood Institute, Bethesda, MD, United States

**Keywords:** Hispanics, Latinos, diabetes, cancer, asthma, obesity, dementia, liver disease

## Abstract

Hispanics/Latinos are expected to constitute 25% of the U.S. population by 2060. Differences in the prevalence of health risk factors, chronic diseases, and access to and utilization of health-care services between Hispanics/Latinos and other populations in the U.S. have been documented. This study aimed to describe and analyze the landscape of Research Program Grants (RPGs) funded by the National Institutes of Health (NIH) between 2008 and 2015 involving Hispanic/Latino health research in six health condition areas—asthma, cancer, dementia, diabetes, liver/gallbladder disease, and obesity—and to identify opportunities for continued research in these areas. Using an NIH internal search engine, we identified new and renewal Hispanic/Latino health RPGs searching for specific Hispanic/Latino identifiers in the Title, Abstract, and Specific Aims. We used descriptive statistics to examine the distribution of funded RPGs by NIH disease-based classification codes for the six health condition areas of interest, and other selected characteristics. The most prominent clusters of research subtopics were identified within each health condition area, and performance sites were mapped at the city level. Within the selected time frame, 3,221 Hispanic/Latino health-related unique RPGs were funded (constituting 4.4% of all funded RPGs), and of those 625 RPGs were eligible for review and coding in the present study. Cancer and obesity were the most commonly studied health condition areas (72%), while studies on mechanisms of disease—biological and non-biological—(72.6%), behavioral research (42.1%) and epidemiological studies (38.1%) were the most common types of research. Most of the primary performance sites were in California, Texas, the northeastern U.S., and Illinois. The predominance of mechanistic, behavioral, and epidemiological studies in our analysis poses opportunities to evaluate knowledge gained and their clinical application, explore new research questions, or to update some methods or instruments. The findings of the present study suggest opportunities to expand research in understudied mechanisms of disease that could explain differences in prevalence of conditions like diabetes and cancer among different heritage groups. In addition, our findings suggest that the impact of interventions or policies designed to reduce health disparities, innovative multi-level interventions, implementation and dissemination studies, the role of health information technology on health outcomes, and the intersectionality of individual, sociocultural, geographic, and other factors on health outcomes, among others, are understudied approaches, which could potentially advance research in Hispanic/Latino health and contribute to the achievement of better health outcomes in this diverse population.

## Introduction

As more precise diagnostic and prognostic algorithms are developed in clinical medicine, understanding the manifestation of health and diseases across age, sex/gender, and racial/ethnic groups has continued gaining critical interest among biomedical researchers. To reach this goal, studies specifically dedicated to understanding patterns of wellness, disease, risk or protective factors (including gene-environment interactions), mechanisms of disease, and effective preventive and treatment strategies, especially among populations affected by health disparities, need to be effectively supported (1–3).

Hispanics/Latinos, the largest U.S. racial/ethnic minority group, are expected in 2060 to constitute a quarter of the U.S. population (4, 5). Differences in access to care and prevalence of chronic diseases between Hispanics/Latinos and other populations in the U.S. have been documented (6–13). Also, differences in prevalence of some chronic diseases or their risk factors among different heritage groups in the contemporary U.S. Hispanic/Latino population have been described by the landmark study, the Hispanic Community Health Study/Study of Latinos (HCHS/SOL) (14–20). However, the underlying mechanisms mediating such differences are yet to be determined. In addition, despite the well-described disproportional burden of health risk factors and adverse social determinants of health, all-cause mortality among U.S. Hispanics/Latinos is lower and their life expectancy is longer for both men and women than other racial/ethnic groups (13, 21–24). The reasons for this health paradox are not fully understood, and further study of the Hispanic/Latino populations may lead to important scientific discoveries.

Motivated by an interest in advancing research in Hispanic/Latino health, the National Institutes of Health (NIH) and the Hispanic Health Research Scientific Interest Group (HHRSIG) (25) reviewed the NIH portfolio of extramural research program grants (RPGs) funded from 2008 to 2015. The goal of this review was to perform a descriptive analysis of funded RPGs in which Hispanics/Latinos had been identified as a population of interest to understand the scope of topics and types of research and identify areas of future research potential. Upon considering the leading causes of death (13, 26–28) as an ethnic group, and the high or increasing prevalence of some chronic diseases (8–20) within the ethnic group or among some Hispanic/Latino heritage groups, this portfolio analysis focused on funded RPGs studying asthma, cancer, dementia, diabetes, liver disease-gallbladder disease (GBD), and obesity.

## Materials and Methods

### Overview of Study Protocol

Extramural staff members of the HHRSIG conducted the portfolio analysis. The analysis team designed a protocol to identify, review, and code select elements of the proposed research in funded RPG applications, as described below. The query/view/report (QVR) is an internal tool that lets NIH extramural staff search, review, and retrieve detailed information about grant applications and awards. QVR was utilized to identify RPG applications funded between fiscal years (FY) 2008 and 2015. This time frame was selected based on a higher accuracy and completeness of the digital database, which tracks starting in 2008 and contained complete data up to 2015 at the time that the portfolio analysis was performed.

The study protocol was pilot-tested and revised before implementation. Applications were randomly distributed among three reviewing teams. A primary and a secondary reviewer were assigned to each application. Disagreements were resolved via team discussion including a third reviewer. The final coding was collected from each team, and the results merged into a single dataset for analysis. The review of applications was initiated in June 2016 and finished in September 2017.

### Definitions

#### RPGs

RPGs constitute most of the annually funded extramural projects (29, 30) and include investigator-initiated projects and those responding to specific NIH initiatives or funding opportunity announcements (FOA) funded through grants. RPGs include the activity codes listed in the NIH Funding—Budget and Spending page (29).

#### Hispanic/Latino Health Research Identifiers

Hispanic/Latino health research was defined as (1) projects that specifically identified Hispanics/Latinos as the population or one of the populations of interest in the U.S. or abroad, and (2) projects in which the recruitment of Hispanics/Latinos in the U.S. or abroad was an intended component of the research plan. Although biomedical research that impacts human health could be performed with animal models or other resources, we defined Hispanic/Latino health research from the perspective of human participants (or their biospecimens or data). To identify projects that specifically identified Hispanics/Latinos as the population or one of the populations of interest in the U.S. or abroad, we used a cluster of 52 Hispanic/Latino search terms that included the Office of Management and Budget (OMB) racial/ethnic categories (31, 32), Latin American nationalities, and other demonyms, countries, capitals, and regions (33–35) (Table 1). Most of these search terms already exist in QVR as NIH Research, Condition, and Disease Category (RCDC) concepts (36), whereas others were added manually. The QVR system would identify these terms throughout the Titles, Abstracts and Specific Aims of the funded RPG applications previously identified. Demonyms, nationalities, and names of countries and capitals or regions were searched in English; no accents or tildes were used.

**Table 1:** Internal search engine Hispanic/Latino identifiers.

**Andean**	**Ecuador, Ecuadorian**	**Mixtec**
Amerindian	El Salvador	Nicaragua, Nicaraguan
Argentina, Argentinian	French Guyana	Panamá, Panamanian
Asuncion	Garifuna	Paraguay, Paraguayan
Aymara	Guadeloupe	Peru, Peruvian
Belize	Guatemala, Guatemalan	Puerto Rico, Puerto Rican
Bolivia, Bolivian	Haiti, Haitian	Quechua
Brazil, Brazilian	Hispanic Americans	Rio de Janeiro
Buenos Aires	Hispanic Community Health Study/Study of Latinos	Salvadoran
Caribbean, Caribbean Hispanics	Hispanic Populations	San Jose (Costa Rica)
Central America	Hispanics	Santiago (Chile)
Central American		
Chicanas, Chicanos	Honduran, Honduras	Santo Domingo
Chile, Chilean	La Paz	São Paulo
Colombia, Colombian	Latina, Latinas, Latino	South America, South American
Costa Rica, Costa Rican	Latino Population	South American Amerinds
Cuba, Cuban	Managua	South American Indians
Cuban Americans	Martinique	Tegucigalpa
Dominican	Mexican, Mexico	Uruguay, Uruguayan
Dominican Republic	Mexican Americans	Venezuela, Venezuelan

#### Health Condition Areas

Health condition areas were defined based on existing RCDC categories and concepts (36). These included: asthma, diabetes, obesity, liver disease (chronic liver disease, cirrhosis, liver disease, hepatitis), GBD (digestive diseases—gallbladder disease), and cancer (all types). For dementia, we used a cluster of established categories and concepts that comprised acquired cognitive impairment, vascular cognitive impairment, dementia, Alzheimer's disease-related dementias, Alzheimer's disease including related dementias, frontotemporal dementia, and Lewy body dementia. We did not modify any of these RCDC categories and concepts.

#### Type of Research

Types of research were defined as basic, mechanistic studies (biological and non-biological), clinical—including epidemiology and clinical trials—and translational research, according to the NIH Glossary and Acronym List (37–41). Health services research, secondary data analyses, and community-based participatory research (CBPR) were defined according to other resources (42–44).

#### Age Groups

Many applications stated the minimum age of study participants (to be enrolled or already enrolled in renewal studies) or the inclusion of children, whereas some stated the specific age range. For simplicity, we created the following age group categories: infants (0–24 months), children (0–17 years), young adults (18–25 years), adults (18–64 years), older adults (65 years and older), and family studies (e.g., dyads or triads of children and parents or guardians).

#### Performance Sites

Primary performance site was defined as the city where the institution receiving the RPG award was located. Secondary performance sites were the cities where institutions subawarded or contracted by the institutions receiving the RPGs were located.

### Study Protocol

#### Selection of RPGs

The identification of eligible RPGs for the analysis involved tiers of search criteria. These criteria included type of application (new or renewal), projects involving Hispanic/Latino health research (as defined by identifiers described above), and projects in health condition areas of interest.

Only new and renewal funded RPG applications were reviewed. RPGs funded by more than one NIH Institute were counted once. RPGs with multiple sites or multi-awards with identical project number were also counted once. In addition, RPG applications were eligible for review based on: identification of Hispanics/Latinos as a population of interest in the Title, Abstract, or Specific Aims; RCDC-based health condition area of interest; recruitment of human research participants (e.g., Hispanics/Latinos), or collecting their data or biospecimens (primary data collection), or analyzing previously collected data or biospecimens (secondary data analysis); availability of an enrollment table in the application; and identifying Hispanics/Latinos in the planned enrollment table.

Non-RPG applications funded through contracts or cooperative agreements (other than U01) or intramural projects were not included in the search or analyses. Any RPGs that were originally funded prior to 2008, even if renewed between FY2008-FY2015, were excluded because both the original applications and planned enrollment tables were not electronically traceable at the time the review and analyses were performed. Interim or final progress reports, final enrollment tables, publications of study findings, or the principal investigators' self-reported race/ethnicity were not reviewed, since these data elements were outside of the scope of this analysis.

#### Other Variables and Categories of Interest

The review of eligible applications involved coding for a set of variables including: health condition area, planned total sample size, planned Hispanic/Latino sample size; incorporation of race/ethnicity into the power calculation of the sample size; Hispanic/Latino heritage group(s) to be represented in the study; age range of participants to be enrolled or already enrolled (for renewals); whether the application was submitted in response to a topic-specific FOA; type of research and performance sites.

Projects were categorized based on whether Hispanic/Latino ethnicity had been accounted for in the power calculation of the planned sample size, as described under the Research Design section of the RPG applications. Projects in which the power calculation had not accounted Hispanic/Latino ethnicity (including those in which ethnicity was only a confounder or effect modifier) were defined as *Planned Enrollment, Not Powered (Not Powered)*. Projects in which the power of the sample calculation accounted Hispanic/Latino ethnicity were defined as *Planned Enrollment, Powered (Powered)*. Projects in which the Specific Aims clearly stated their focus was exclusively on Hispanics/Latinos or whose projected sample size was 100% Hispanic/Latino (independent of the heritage group) were defined as *Focused*. These categories were applied whether the studies involved primary recruitment/collection of human research participants/data or secondary analyses of biospecimens or data. We did not evaluate whether the power of the planned sample size was adequately estimated based on race/ethnicity or to address the proposed research questions.

### Analyses

The descriptive analysis was performed using SAS version 9.4 (Cary, NC). Statistical tests were carried out using the non-parametric Mann-Whitney-Wilcoxon rank sums test to compare some of the variables (e.g., sample size by power category, and by new planned enrollment compared to existing cohorts/datasets). Since some RPG applications involved more than one health condition area and most involved more than one type of research (explained under Results), comparisons of these variables were not performed. Performance sites were also mapped at the city level using SAS.

The frequency and grouping of research subtopics within each health condition area were analyzed using text mining and language processing methods. Within each health condition area, the selected RPGs were further clustered by subtopics using *iSearch v2.4* a suite of applications for portfolio analyses created by the NIH Office of Portfolio Analysis (45), and only accessible to NIH staff. Within *iSearch* Grants Module, the Visualize Results tool uses a clustering algorithm that uses words and phrases from the Title, Abstract, and Specific Aims of grants to create “foam trees.” Titles are given more weight in the clustering algorithm. Before clustering, the Title, Abstract, and Specific Aims of each selected RPG were preprocessed using stemming, stop words, and synonym normalization. The clusters displayed were scaled to the number of grant applications.

## Results

**Figure 1:**
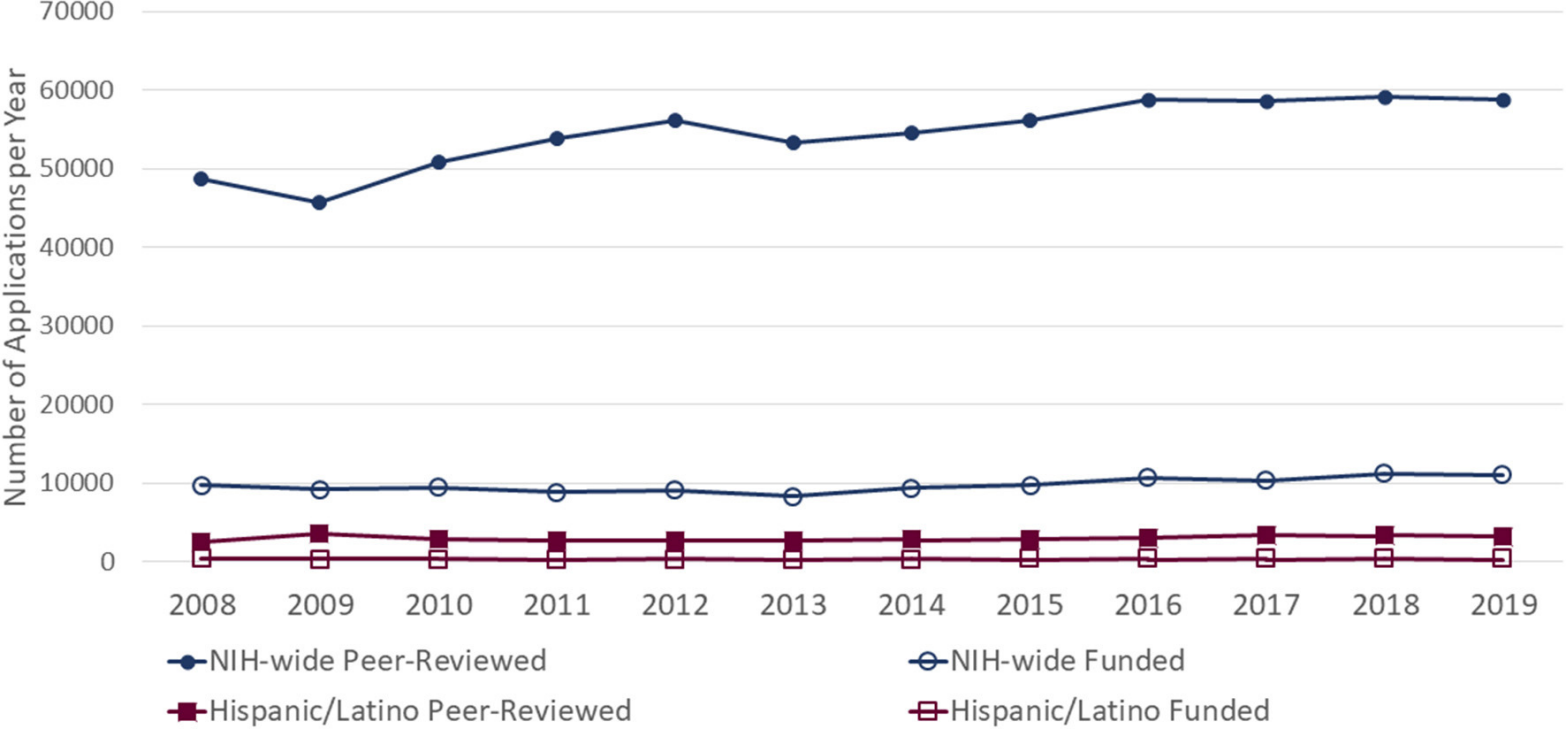
Peer-reviewed and funded New and Renewal RPG applications, 2008–2019.

From FY2008 to FY2015, a total of 3,221 Hispanic/Latino health-related unique RPGs were funded, and of those, 625 RPGs were eligible for review and coding in the present study. Figure 1 shows the numbers of peer-reviewed and funded new and renewal RPG applications both NIH-wide and involving Hispanic/Latino health. From FY2008 to FY2015, over 450,000 new and renewal RPG applications were reviewed by study sections, and ~18.0% were funded every year, which is congruent with NIH funding rates and success rates reports (30, 46). Among the peer-reviewed RPGs, QVR identified Hispanic/Latino search terms in the Titles, Abstracts, or Specific Aims of 5.3% of the applications. Among these, 3,221 unique new or renewal applications were funded (representing 14.9% of the Hispanic/Latino peer-reviewed pool and 4.4% the NIH-wide funded RPG pool). Similar reviews and funding trends are observed from FY2016 to FY2019.

**Figure 2:**
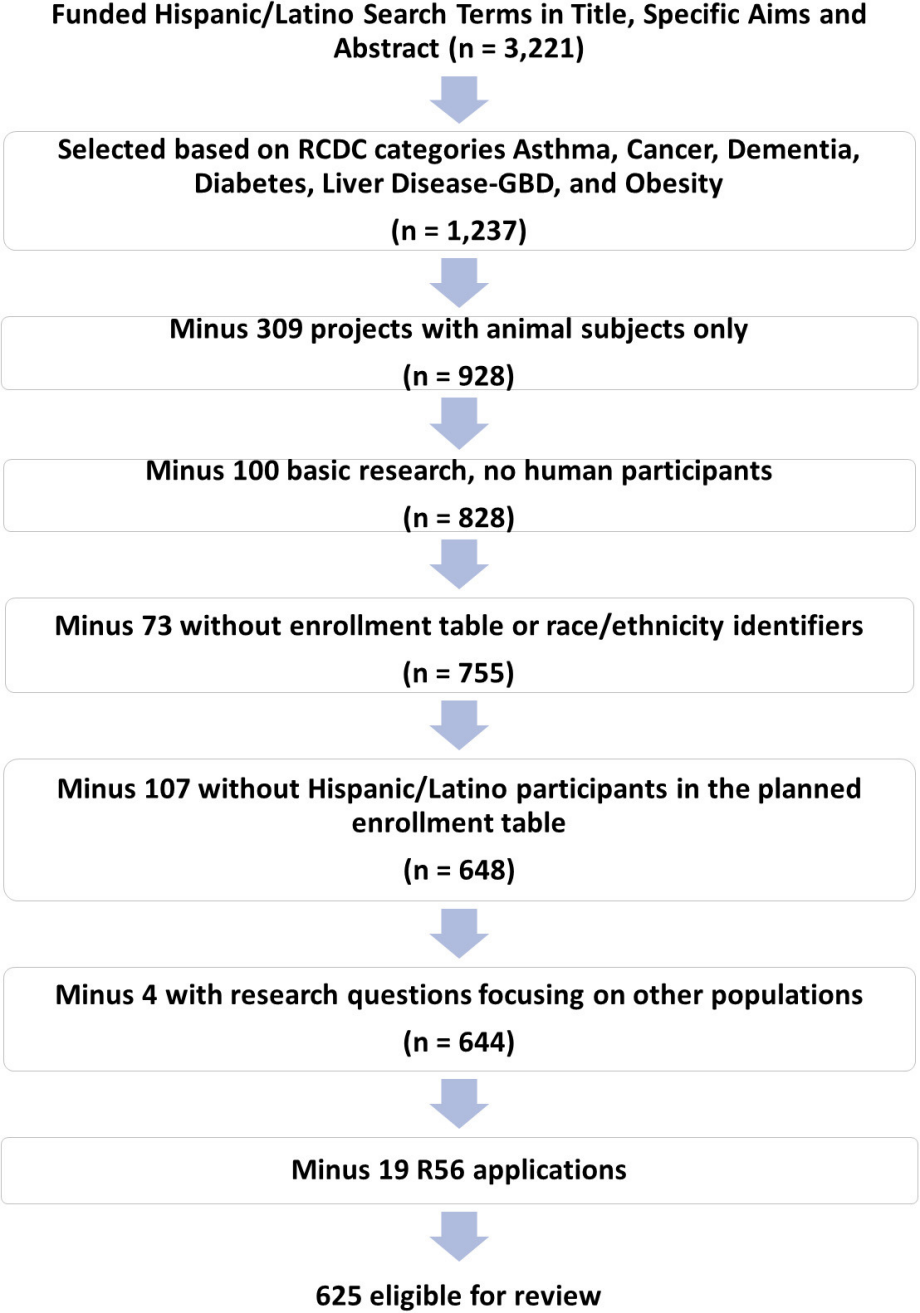
Selection of RPGs and review eligibility criteria.

Among the total 3,221 funded RPGs studying Hispanic/Latino health, 1,237 addressed at least one of the six health condition areas of interest (Figure 2). Of these, 612 were excluded from the analysis for the following reasons: 309 included animal subjects only; 100 involved basic research and did not describe either animal or human research subjects; 73 did not include an enrollment table or race/ethnic breakdown of participants; and 107 did not mention Hispanic/Latinos in the planned enrollment table. In addition, four (4) projects explicitly mentioned Hispanics/Latino in the proposed enrollment table, but the main hypotheses were focused on other populations and Hispanics/Latinos were not mentioned in the statistical plan. We also decided to not review R56 (bridge) awards (*n* = 19) since they did not incorporate new enrollment of participants or major changes in study activities compared to the parent grant. Although the NIH Annual Fiscal Year Funding Report does not include American Recovery and Reinvestment Act (ARRA) awards, we included 18 ARRA-awarded applications addressing Hispanic/Latino research in the review. Therefore, a total of 625 applications were coded and comprised the analytical dataset.

### Types of Research and Health Condition Areas

Although 625 unique RPGs were coded, some of the RPGs fell within more than one RCDC category. Table 2 illustrates the breakdown of the coded RPGs by health condition area. Applications with more than one RCDC category included: Asthma and Obesity (1), Cancer and Liver Disease-GBD (1), Cancer and Obesity (1), Dementia and Diabetes (1), Dementia and Diabetes and Obesity (1), Diabetes and Liver Disease-GBD (3), Diabetes and Liver Disease-GBD and Obesity (2), Diabetes and Obesity (45), Liver Disease-GBD and Obesity (2), and Obesity and Dementia (1). Upon counting for these, the number of applications by health condition area added up to 686. Most applications (89.8%) were new projects, 73% focused on Cancer and Obesity, and 83% of the applications were funded through R01, R03, and R21 grants. Overall, slightly more than half of the applications were solicited (responded to topic-specific FOAs). Studies involving mechanisms of disease (72.6%), behavioral components (42.1%), and epidemiology (38.7%) were the most predominant types of research and overlapped, especially across asthma, diabetes, and obesity health condition areas (data not shown). Translational, CBPR, and health services research were the least common type of research. Also, 191 clinical trials were identified, of which 54% tested behavioral interventions (data not shown). Phase III clinical trials made up just 4% of the coded RPGs (*n* = 26) (data not shown).

**Table 2:** Overview of funded Hispanic/Latino health RPGs by Health Condition Area.

	**Asthma**	**Cancer**	**Dementia**	**Diabetes**	**Liver Disease-GBD**	**Obesity**	**Unique applications**
N^*^	45	273	37	123	27	181	625
**Type of Application^#^**							
New	41 (91.1)	245 (89.7)	30 (81.1)	108 (87.8)	19 (70.4)	169 (93.4)	561 (89.8)
Renewals	4 (8.9)	28 (10.3)	7 (18.9)	15 (12.2)	8 (29.6)	12 (6.6)	64 (10.2)
**Activity Code^#^**							
R01	24 (53.3)	124 (45.4)	20 (54.1)	72 (58.5)	14 (51.9)	100 (55.2)	314 (50.2)
R03/R21	12 (26.7)	107 (39.2)	9 (24.3)	26 (21.1)	4 (14.8)	59 (32.6)	208 (33.3)
Other R	2 (4.4)	3 (1.1)	0 (0.0)	6 (4.9)	2 (7.4)	7 (3.9)	15 (2.4)
U01	2 (4.4)	25 (9.2)	4 (10.8)	13 (10.6)	7 (25.9)	12 (6.6)	57 (9.1)
Other U	2 (4.4)	5 (1.8)	0 (0.0)	2 (1.6)	0 (0.0)	0 (0.0)	9 (1.4)
ARRA	3 (6.7)	8 (2.9)	2 (5.4)	4 (3.3)	0 (0.0)	2 (1.1)	18 (2.9)
Other	0 (0.0)	1 (0.4)	2 (5.4)	0 (0.0)	0 (0.0)	1 (0.6)	4 (0.6)
RFA/PAR/PA solicited^#^	19 (42.2)	176 (64.2)	18 (48.6)	71 (57.7)	14 (51.9)	94 (51.9)	360 (57.8)
**Type of Research (%)^¶^**							
Basic research	33.3	36.6	10.8	26.8	37	12.2	26.7
Mechanisms of disease	73.3	72.2	70.3	65.9	88.9	76.2	72.6
Epidemiology	40.0	37.0	51.4	37.4	55.6	36.5	38.7
Clinical trials	26.7	27.8	13.5	33.3	29.6	39.8	30.6
Behavioral	28.9	36.3	29.7	35.0	37.0	61.3	42.1
Health services research	8.9	16.8	8.1	9.8	0.0	2.8	10.9
Translational research	13.3	17.2	13.5	12.2	7.4	6.6	12.5
CBPR	20.0	13.2	0.0	5.7	7.4	14.4	12.0
Secondary analysis only	31.1	35.9	29.7	37.4	18.5	25.4	32.3

*For N, Type of Application, Activity Code and RFA/PAR/PA Solicited rows, numbers under the All column represent the total and breakdown based on the 625 unique RPGs, and numbers under the disease category columns add up to 686 since some studies were accounted in more than one RCDC disease category*.

* *Results shown in numbers*.

# *Results shown in numbers, and percentage in parentheses*.

¶ *Results shown in percentage. The sum under the “All” column exceeds 100 percent, since some studies were coded in more than one category of type of research*.

Although outside of the scope of our analysis, information on the self-reported Hispanic/Latino ethnicity of the principal investigators of the projects included in the analysis was requested to the NIH Office of Extramural Research (OER) following an established protocol. The data were analyzed by OER and provided in clusters by disease area, not individual answers. Among the 625 RPGs reviewed in this analysis, ~12% of the principal investigators self-reported Hispanic/Latino ethnicity (data not shown). These data were not further analyzed.

### Common Research Themes Within Health Condition Areas

**Figure 3:**
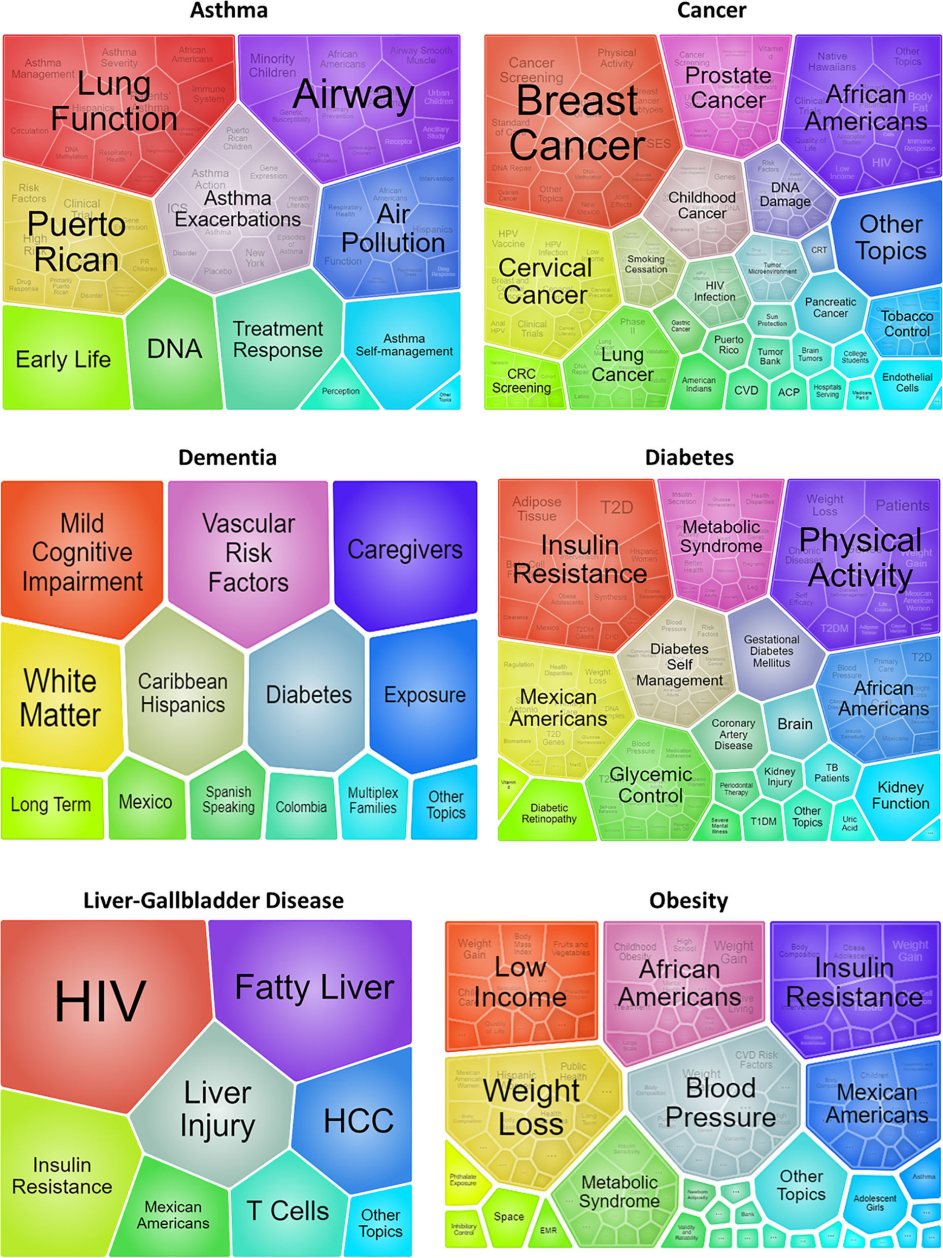
Clusters of research subtopics and cross-cutting themes by Health Condition Area. Each foam tree shows clusters of subtopics within each Health Condition Area scaled to the number of grants.

Within each health condition area, research subtopics were clustered by number of grants (Figure 3 foam tree panels). Projects could have addressed more than one subtopic within each health condition area.

In the Asthma area, the five largest clusters of subtopics included lung function, airway inflammation and reactivity, asthma exacerbations, air pollution (environmental exposures), and studies focused on Puerto Ricans. Studies involving DNA methylation (e.g., epigenetics) and/or gene expression were present within each of these large clusters. Studies involving interventions, especially with dyads (*Family studies* in Table 3), or in which African Americans or school-age children were populations of interest within some of the largest clusters.

**Table 3:** General descriptors of funded RPGs by Health Condition Area and accounting for Hispanic/Latino ethnicity on the power of the planned sample size.

	**Asthma**	**Cancer**	**Dementia**	**Diabetes**	**Liver Disease-GBD**	**Obesity**	**All**
**Planned Hispanic/Latino Enrollment, Not Powered (Hispanic/Latino ethnicity not accounted in the power of the planned sample calculation)**							
N^*^	25	172	18	61	17	99	365
**Planned Hispanic/Latino Sample Size**							
Primary data collection^¶^ (N)	15	109	11	38	15	73	241
Min	7/9.1	1/0.5	9/9.8	2/1.3	7/7.8	3/0.8	1/0.5
Max	715/92.0	8,650/97.3	2,719/56.8	12,500/93.0	800/90.0	14,400/90.0	14,400/97.3
Median	143/41.8	34/15.0	50/34.4	50/23.6	201/40.0	100/37.5	72/26.3
Secondary analyses only^¶^ (N)	10	63	7	23	2	26	124
Min	10/13.1	2/0.6	74/0.8	21/2.5	700/5.2	11/1.2	2/0.6
Max	8,976/97.1	6,044,176/62.8	40,265/33.0	34,513/94.8	2,171/25.7	2,600/85.6	6,044,176/97.1
Median	2,835/59.2	388/13.2	2,319/8.9	2,072/20.1	1,436/15.4	1,602/23.6	934/16.9
**Age Group^#^**							
Infants (0–24 months)	4 (16.0)	35 (20.3)	2 (11.1)	4 (6.6)	3 (17.6)	4 (4.0)	52 (14.2)
Children (0–17 years)	8 (32.0)	10 (5.8)	1 (5.6)	6 (9.8)	3 (17.6)	29 (29.3)	50 (13.7)
Children (0–17 years) and Young adults (18–25 years)	4 (16.0)	2 (1.2)	0 (0.0)	2 (3.3)	1 (5.9)	4 (4.0)	10 (2.7)
Adults (25–64 years)	3 (12.0)	94 (54.7)	4 (22.2)	32 (52.5)	9 (52.9)	30 (30.3)	160 (43.8)
Adults, Older Adults (65+)	0 (0.0)	20 (11.6)	11 (61.1)	15 (24.6)	1 (5.9)	14 (14.1)	56 (15.3)
Family study	6 (24.0)	11 (6.5)	0 (0.0)	1 (1.6)	0 (0.0)	18 (18.2)	36 (9.9)
Not specified	0 (0.0)	0 (0.0)	0 (0.0)	1 (1.6)	0 (0.0)	0 (0.0)	1 (0.3)
**Proposed Hispanic/Latino Enrollment, Powered (Hispanic/Latino ethnicity accounted in the power of the planned sample calculation)**							
N	5	42	3	15	2	19	78
**Planned Hispanic/Latino Sample Size**							
Primary Data Collection^¶^ (N)	2	20	3	5	1	10	39
Min	100/38.8	17/1.2	80/50.0	28/33.3	28/33.3	28/15.0	17/1.2
Max	155/66.7	47,438/96.3	366/83.3	1,000/50.0	28/33.3	2,312/94.9	47,438/96.3
Median	128/52.7	262/50.0	250/73.2	120/33.3	28/33.3	318/46.3	278/50.0
Secondary Data Analyses Only^¶^ (N)	3	22	0	10	1	9	39
Min	500/48.0	100/3.6	NA	748/10	47,438/22.0	748/10.0	100/3.6
Max	5,900/66.3	47,438/59.3	NA	334,298/66.7	47,438/22.0	47,438/64.1	334,298/66.7
Median	4,800/50.0	2,000/26.2	NA	36,166 (28.6)	47,438/22.0	2,002/22.0	14,675/26.7
**Age Groups^#^**							
Infants (0–24 months)	0 (0.0)	2 (4.8)	0 (0.0)	2 (13.3)	0 (0.0)	2 (10.5)	4 (5.1)
Children (0–17 years)	3 (60.0)	3 (7.1)	0 (0.0)	1 (6.7)	0 (0.0)	5 (26.3)	11 (14.1)
Children (0–17 years) and Young adults (18–25 years)	2 (40.0)	3 (7.1)	0 (0.0)	1 (6.7)	1 (50.0)	1 (5.3)	6 (7.7)
Adults (25–64 years)	0 (0.0)	20 (47.6)	2 (66.7)	8 (53.3)	0 (0.0)	2 (10.5)	32 (41.0)
Adults, Older adults (65+)	0 (0.0)	11 (26.2)	1 (33.3)	1 (6.3)	1 (50.0)	4 (21.2)	16 (20.5)
Family study	0 (0.0)	3 (7.1)	0 (0.0)	1 (6.7)	0 (0.0)	5 (26.3)	8 (10.3)
Not specified	0 (0.0)	0 (0.0)	0 (0.0)	1 (6.7)	0 (0.0)	0 (0.0)	1 (1.3)
**Focused (Planned sample was 100% Hispanic/Latino or studies focused on Hispanics/Latinos)**							
N^*^	15	59	16	47	8	63	182
**Planned Hispanic/Latino Sample Size**							
Primary Data Collection^¶^ (N)	14	46	12	34	6	52	143
Min	20/19.7	28/50.0	70/100.0	20/100.0	56/100.0	20/96.2	20/19.7
Max	4,000/100.0	6,000/100.0	6,539/100.0	2,200/100.0	500/100.0	4,014/100.0	6,539/100.0
Median	267/100.0	313/100.0	400/100.0	300/100.0	268/100.0	306/100.0	300/100.0
Secondary Data Analyses Only^¶^ (N)	1	13	4	13	2	11	39
Min	4,343/82.2	20/100.0	1,789/100.0	300/100.0	407/100.0	700/100.0	20/82.2
Max	4,343/82.2	6,800/100.0	8,809/100.0	5,638/100.0	2,539/100.0	230,000/100.0	230,000/100.0
Median	4,343/82.2	1,302/100.0	2,836/100.0	2,186/100.0	1,473/100.0	1,272/100.0	1,725/100.0
**Age Groups^#^**							
Infants (0–24 months)	1 (6.7)	6 (10.2)	0 (0.0)	4 (8.5)	1 (12.5)	3 (4.8)	11 (6.0)
Children (0–17 years)	7 (46.7)	7 (11.9)	0 (0.0)	9 (19.1)	2 (25.0)	8 (12.7)	31 (17.0)
Children (0–17 years) and Young adults (18–25 years)	3 (20.0)	0 (0.0)	0 (0.0)	2 (4.3)	0 (0.0)	5 (7.9)	8 (4.4)
Adults (25–64 years)	0 (0.0)	32 (54.2)	5 (31.3)	26 (55.3)	5 (62.5)	32 (50.8)	86 (47.3)
Adults, Older adults (65+)	0 (0.0)	12 (20.3)	11 (68.8)	5 (10.6)	0 (0.0)	7 (11.1)	31 (17.0)
Family study	4 (26.7)	1 (1.7)	0 (0.0)	0 (0.0)	0 (0.0)	8 (12.7)	13 (7.1)
Not specified	0 (0.0)	1 (1.7)	0 (0.0)	1 (2.1)	0 (0.0)	0 (0.0)	2 (1.1)

*Values under the “All” column represent the total and breakdown based on the 625 unique RPGs, and values under the disease category columns add up to 686 (392 Not-Powered, 86 Powered, and 208 Focused) since some studies were accounted in more than one RCDC disease category*.

* *Results shown in numbers. The numbers presented by disease category and power of the sample add up to more than the total number in Table 1 because some studies were coded in more than one RCDC disease category*.

# *Results shown in numbers, and percentage in parentheses*.

¶ *The proposed Hispanic/Latino sample size is shown as total number of Hispanic/Latino research participants and percentage from the total sample. The number and the percentage represent two separate distributions and do not coincide with each other*.

In the Cancer area, the five largest clusters of subtopics involved breast, cervical, prostate, lung, and childhood cancers. Genetics/genomic studies were common among each cluster, and especially under lung and childhood cancer. Quality of care, socioeconomic status, or barriers and strategies to increase or facilitate cancer screening were also common topics addressed across the largest clusters.

In the Dementia health condition area, the larger clusters of subtopics were almost evenly distributed, but the three largest clusters involved mild cognitive impairment (including screening), vascular risk factors, and studies (interventions) involving caregivers.

Within the Diabetes area, the largest clusters of subtopics by number of grants involved insulin resistance and physical activity, followed by Mexican Americans, African Americans, metabolic syndrome, self-management, and glycemic control. Studies in which Mexican Americans or African Americans were populations of interest were prominent and represented in various clusters. Genetic research was prominent in the physical activity cluster.

The Liver-GBD area had the smallest number of RPGs, and the largest clusters of subtopics involved HIV and fatty liver, which included non-alcoholic fatty liver disease (NAFLD) or non-alcoholic steatohepatitis (NASH).

Within the Obesity area, the largest clusters of subtopics included studies on insulin resistance, weight loss, blood pressure, Mexican Americans, African Americans, and the metabolic syndrome. Studies on body composition, children, adipose tissue, and genetic/genomic studies were common across these large clusters. Mexican Americans and African Americans were populations of interest across multiple subtopics, especially those involving weight loss and control of cardiovascular risk factors.

In a preliminary overview of the clusters of topics for RPGs funded in 2016–2019, similar groups of subtopics within each health condition area areas were observed (data not shown).

### Hispanic/Latino Ethnicity in the Sample Calculation

Forty-two percent (42%) of the coded RPGs incorporated Hispanic/Latino ethnicity in the power calculation of the sample size or focused exclusively on Hispanics/Latinos. Table 3 shows the breakdown of coded RPGs by health condition area and accounting of Hispanic/Latino ethnicity into the sample size calculation. The distribution of types of RPGs was similar across power categories, except that U01 applications were more common within the Not-Powered category (data not shown). Hispanic/Latino sample size of the Focused studies were significantly larger than those of the Not Powered studies (*P* < 0.001), while no difference was observed for total number of participants (*P* = 0.115). Planned sample sizes were subcategorized into primary recruitment/data collection and secondary analyses. Most studies (66% Not Powered, 50% Powered, and 78% Focused) involved primary recruitment and/or data collection. Secondary analyses had overall median planned Hispanic/Latino sample size consistently larger than studies involving primary recruitment/data collection (*P* < 0.001). Independent of the power category, most of the studies on asthma focused on children and young adults, most cancer and diabetes studies focused on adults aged 25–64 years, studies on liver/gallbladder disease and obesity covered different ages, and most of the dementia studies focused on adults aged 25–64 years and older adults.

### Performance Sites

**Figure 4:**
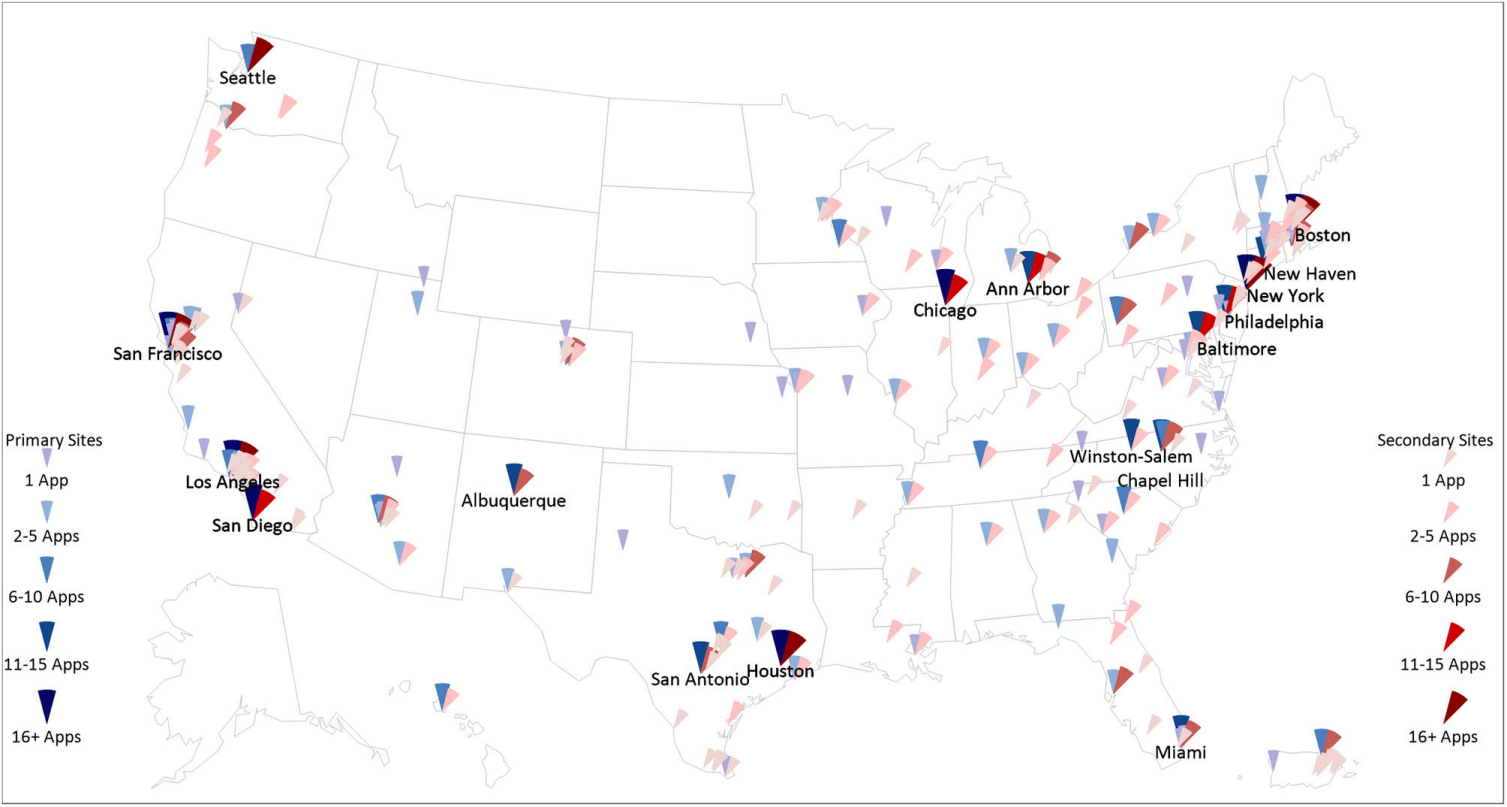
Primary and secondary U.S. performance sites of coded Hispanic/Latino RPGs.

Primary performance sites predominated in California (*n* = 142), Texas (*n* = 64), the Northeast [New York (*n* = 59), and Massachusetts (*n* = 41)], Illinois (*n* = 26), and Florida (*n* =22). Arizona (*n* = 16), New Mexico (*n* = 12), Colorado (*n* = 9) and Puerto Rico (*n* = 7) and had the lowest number of primary performance sites (Figure 4). Secondary performance sites predominated in California (*n* = 118), Texas (*n* = 57), New York (*n* = 53), and Massachusetts (*n* = 52).

In addition, 45 of the coded RPGs planned to enroll participants or perform the studies in Latin America and represented 7 primary and 38 secondary performance sites (data not shown). Most of these sites were in Mexico (3 primary and 21 secondary sites), while others were in Argentina, Brazil, Chile, Colombia, Costa Rica, Dominican Republic, Guatemala, Honduras, Peru, Uruguay, and Venezuela. Twenty-six (26) of the RPGs in Latin America focused on Cancer, 11 on Diabetes and/or Obesity, 5 on Dementia, and 2 on Asthma. Also 20 (43.5%) of the RPGs proposed studies of mechanisms of disease, 17 (37%) basic research, 19 (41.3%) epidemiology, 15 (32.6%) behavioral studies, 13 (28.2%) clinical trials, and 4 (8.7%) secondary data analyses (data not shown).

### Identification of Hispanic/Latino Populations of Interest in the Proposed Research Projects

**Figure 5:**
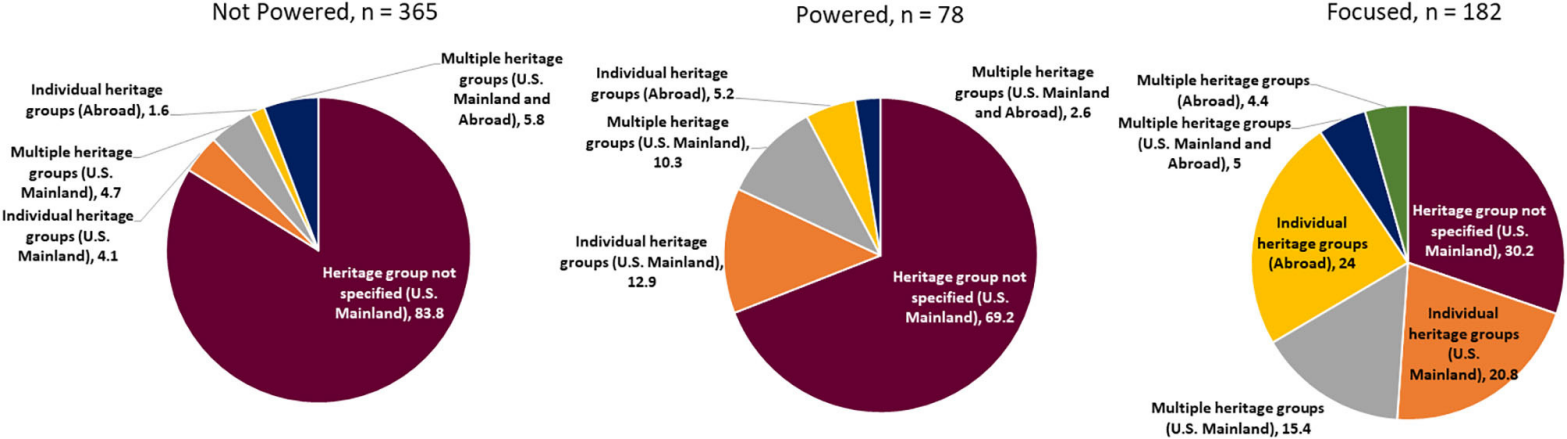
Hispanic/Latino heritage groups of interest by sample power category.

Overall, 67% of applications used the term “Hispanic,” “Latino,” or “Spanish speakers” to describe the population of interest and did not further specify a Hispanic/Latino heritage group. Figure 5 shows how investigators described Hispanic/Latino populations of interest in the U.S. and abroad by sample/power category. Whereas, most of the Not Powered studies used the umbrella terms “Hispanics” or “Latinos,” most of the focused studies identified individual Hispanic/Latino heritage groups of interest. Sixty-seven (67) projects specified heritage groups of interest to be recruited in the U.S. Mainland: Mexican (13 Not Powered, 9 Powered, 30 Focused), Puerto Rican (2 Not Powered, 2 Powered, 2 Focused), Dominican (3 Not Powered, 1 Powered, 4 Focused), and Haitian (1 Focused) (data not shown). Forty-nine (49) projects identified specific heritage groups of interest to be recruited outside of the U.S. Mainland: Mexican (1 Not Powered, 1 Powered, 10 Focused), Puerto Rican (11 Focused), Central American (5 Focused), and South American (2 Not Powered, 2 Powered, 17 Focused) (data not shown). Across sample/power categories, Mexican was the most common individual Hispanic/Latino heritage group of interest of studies.

### Studies Not Included in the Analysis

As described under Methods, RPGs that were originally funded prior to FY2008, even if renewed between FY2008-FY2015, were excluded because both the original applications and enrollment tables were not traceable. Similarly, non-RPGs funded prior to FY2008 or during the selected timeframe were not included. Table 4 shows RPGs funded prior to FY2008 and non-RPGs funded prior to and during FY2008, which were not included in the analysis but addressed any of the six health condition areas of interest and in which Hispanics/Latinos were the focus or one of the populations of interest or recruited (14–20, 47–80).

**Table 4:** Non-RPG studies in the six Health Condition Areas funded before or during FY2008-FY2015 with recruitment of Hispanics/Latinos in the U.S and/or Abroad.

Action to Control Cardiovascular Risk in Diabetes (ACCORD) Study^#^ (47) Boricua Youth Study^**^^§^ (48) Boston Puerto Rican Health Study^**^^§^ (49) Corpus Christi Heart Project^*^^§^ (50) Diabetes Control and Complications Trial (DCCT)/Epidemiology of Diabetes Interventions and Complications (EDIC)^#^ (51, 52) Diabetes Prevention Program (DPP)/Diabetes Prevention Program Outcomes Study (DPPOS)^#^ (53) Environmental Influences on Child Health Outcomes (ECHO) Program^#^ (54) Gastroparesis Clinical Research Consortium (GpCRC)^#^ (55) Genetics of Asthma in Latino Americans (GALA) Study^**^^§^^†^ (56) Glycemia Reduction Approaches in Diabetes (GRADE) Study^#^ (57) Healthy Communities Study^#^ (58) HEALTHY study^#^ (59) Hispanic Community Health Study/ Study of Latinos (HCHS/SOL)^#^^§^^†^ (14–20) Hispanic Established Populations for Epidemiologic Studies of the Elderly (Hispanic EPESE)^#^ ^§^ (60) Hyperglycemia and Adverse Pregnancy Outcome (HAPO) Study^#^ (61) Insulin Resistance Atherosclerosis Study (IRAS)^#^ (62) Look AHEAD (Action for Health in Diabetes) Study^#^ (63) The Multiethnic Cohort (MEC) Study^##^ (64) Multi-Ethnic Study of Atherosclerosis (MESA)^#^^§^^†^ (65) NHLBI-UnitedHealth Global Health Initiative^#^^§^^†^ (66) Non-alcoholic Steatohepatitis Clinical Research Network (NASH CRN)^#^ (67) Patterns of Care Studies^#^ (68) Puerto Rico Heart Health Program^#^^§^ (69) Restoring Insulin Secretion (RISE) Study^#^ (70) Sacramento Area Latino Study on Aging (SALSA)^**^^§^ (71) San Antonio Heart Study^**^^§^ (72) San Antonio Longitudinal Study on Aging (SALSA)^**^^§^ (73) San Luis Valley Diabetes Study^*^^§^ (74, 75) SEARCH for Diabetes in Youth Study^#^ (76) The Environmental Determinants of Diabetes in the Young (TEDDY) study^#^ (77) Treatment Options for Type 2 Diabetes in Adolescents and Youth [TODAY] Study^#^ (78) Vitamin D and Type 2 Diabetes (D2d) Study^#^ (79) Women's Health Initiative (WHI)^#^ (80)

* *Original RPG funded before the reviewed time frame*.

** *Original RPG funded before the reviewed time frame; renewal(s) or ancillary studies included in the analysis*.

# *Non-RPG funded before or during the reviewed time frame*.

## *Original non-RPG funded before or during the reviewed time frame; renewal(s) or ancillary studies included in the analysis*.

§ * Hispanics/Latinos were enrolled and the focus of the study*.

† *Different Hispanic/Latino heritage groups were identified*.

## Discussion

In 1940, photographer Jack Delano was employed by the Farmers Security Administration (FSA) to photograph not only farmers, but all aspects of current social conditions in the U.S Virgin Islands and Puerto Rico. In the introduction to his book *Puerto Rico M*í*o* (81), Delano recalls a letter from a 14-year-old boy from El Paso, Texas. The young boy shared his admiration for the FSA pictures that Delano had taken and asked for his autograph. Compelled by the young boy's personal story of poverty and interest in photography, Delano sent him an autographed picture and asked him “What is it that you like about the FSA pictures?” The young boy wrote back: “I like the pictures because they make ordinary people important.”

The present study is a picture, a snapshot of the NIH-funded RPGs on Hispanic/Latino health. Our analysis demonstrated that 4.4% of RPGs funded between FY2008 and FY2015 identified Hispanics/Latinos as a population or the main population of interest. Among the RPGs addressing any of the health conditions of interest, mechanistic, behavioral, and epidemiological studies were the most common type of research, over 70% of the studies involved Obesity or Cancer as their main health condition of interest, 42% proposed a powered sample size or focused on Hispanics/Latinos, and the majority of the performance sites were located in U.S. areas with the highest concentration of Hispanic/Latino population.

The percentage of funded RPGs in which Hispanics/Latinos were a population of interest identified through our methods warrants consideration. The number of funded RPGs identified by our analysis paralleled the number of peer-reviewed RPG applications during the selected time frame. Approximately 12% of the principal investigators in the RPGs included in our analysis self-reported Hispanic/Latino ethnicity. In a previously published NIH-based analysis (82), the percent of NIH R01/RPG-awarded Hispanic/Latino investigators from FY2009 to FY2016 ranged from 3.4 to 5.0%. Although these two analyses evaluated slightly different portfolio metrics, both suggest the previously documented need for greater diversity in the biomedical workforce (83–89), and specifically for promoting the career development of more Hispanic/Latino biomedical researchers. Future studies could assess whether increasing the number of funded Hispanic/Latino investigators may increase the number of RPGs focused on Hispanic/Latino health research.

The types of research identified by our analysis need to be examined through the lens of the progressive recognition and increase in prevalence of chronic diseases and health risk factors and persistent health disparities experienced by many Hispanic/Latino communities. The predominance of mechanistic, behavioral studies (which could have included intervention or retrospective analyses) and epidemiological studies presents opportunities to evaluate knowledge gained and its clinical application, to test newly generated research hypotheses refocus scientific questions (90), update, or integrate novel research methods redesign (91, 92) that connect science with the health profile and health-care needs of the contemporary Hispanic/Latino populations.

At the same time, our analysis suggests that health services research, translational medicine, and/or CBPR are types of research that could be further developed within the context of the six health condition areas that were reviewed. Whereas the focus on implementation science and translational research at the NIH has been increasing (92, 93), our findings suggest that future research could focus on understudied approaches in Hispanic/Latino health, including, but not limited to, deeper understanding of effectiveness of currently recommended therapies and potential differences among heritage groups (56, 94–96); participation and/or increased inclusion in genetics/genomic studies (97–99); innovative strategies to implement recommended guidelines of care, and especially those move beyond the “sideways” approach (100); the intersection (101–103) of social determinants of health other factors on disease risk and the effectiveness of clinical or multi-level interventions (104–107); design and analysis of multi-level or multi-sectoral (108–110) interventions; implementation and dissemination studies in real-world settings (92, 111); the role of health information technologies on health-care delivery and health outcomes (112–118); the impact of interventions or policies designed to reduce health and health-care disparities (119–126); and the effects of national or local policies on health-care services and health outcomes (e.g., natural experiments) (127, 128) among Hispanic/Latino populations.

Although a literature review would offer a comprehensive view of the six health condition areas, the present analysis suggests some opportunities where research in Hispanic/Latino health could be expanded. For example, while asthma tends to be more prevalent in children, it is also highly prevalent in Puerto Rican adults as described in the HCHS/SOL (15). The study of asthma in adults could contribute to further understanding mechanisms of disease and response to treatment, especially in adults with coexisting chronic conditions. Also, most of the asthma interventions/clinical trials (67%) were focused on behavior (data not shown), suggesting that expanding research to other types of interventions could be considered. Many studies on cancer focused on increasing individual and/or community screening, awareness of risk factors, and prevention. Since cancer type and mortality vary by Hispanic/Latino heritage group (26–28, 129), additional research could focus on understanding mechanisms of disease, response to treatment, and risk or protective factors across heritage groups. Research in dementia could expand in multiple dimensions, from novel or unique mechanisms of cognitive decline (130), understanding differences in risk (131, 132) or manifestations of disease, and potential interventions to prevent the disease or delay its progression. Many diabetes studies in our analysis included clinical interventions focused on prevention (e.g., weight loss). The prevalence of diabetes mellitus among U. S. Hispanics/Latinos as a sole ethnic group has been consistently higher than NHWs (9), and varies by heritage group (17). Conversely, Hispanics/Latinos with diabetes mellitus are at increased risk of preventable acute and chronic complications (133, 134) and hospital readmissions (135, 136), and diabetes mellitus is one of the leading causes of death in these populations (137–139). Research on mechanisms underlying these inter-heritage differences, and on implementation of diabetes guidelines of care (140, 141), could potentially generate more targeted interventions that effectively prevent diabetes and its complications. A significant number of liver disease studies focused on hepatitis C within the context of HIV/AIDS, and risk and long-term consequences of liver injury, which are highly relevant among some Hispanic/Latino communities (142–144). Since gallbladder disease is highly prevalent among Hispanics/Latinos (11, 12, 145), and it has been associated with cardiometabolic disease risk factors (146–148), research dedicated to its prevention could impact not only Hispanics/Latinos, but other populations (149–151). Although outside of the scope of our analysis, factors mediating the lower mortality rates documented for Hispanics/Latinos as a group (23, 24, 139, 152, 153) are still not fully understood.

Forty-two percent of the projects included in the analysis focused on Hispanics/Latinos or incorporated Hispanic/Latino ethnicity into the planned sample size power estimates. The incorporation of Hispanic/Latino ethnicity in the power estimation of the planned sample size was an indicator of a deliberate interest to enroll Hispanics/Latinos in the study. In some studies, correlation analyses were proposed when Hispanic/Latino ethnicity was not accounted in the calculation of the power of the proposed sample (data not shown). Although it is not always possible to estimate the power of the planned sample size based on race/ethnicity (154), including a rationale for not doing so and acknowledging the limitations of correlation analyses in answering the central research question could be considered.

In 67% of the RPGs included in our analysis the ethnic identification of prospective participants (and/or data and biospecimen resources) was limited to the general Hispanic or Latino terms. Of note, 73 RPGs were not included in the analysis because the source of data or materials provided no race/ethnicity information. Including other heritage descriptors (e.g., birthplace, heritage or family roots, preferred language, parents' place of birth) could enhance the interpretation of study findings (155–157), since differences in the prevalence of some chronic diseases have been documented among different Hispanic/Latino heritage groups (13–15, 17). Socioeconomic descriptors (e.g., education attained, income) could also enhance the interpretation of health outcomes analyses, which would also be applicable to studies dedicated to other minority and underserved groups. Although confidentiality and data-sharing parameters might need to be evaluated, the collection of a minimum of such identifiers by biobanks, administrative databases, or registries would magnify the contribution of those unique resources to mechanistic studies, including genetics/genomics and precision medicine and health services research (158).

The location and distribution of the primary and secondary performance sites in the U.S. parallels the location of large academic centers and some of the states and cities with the highest concentration of Hispanics/Latinos (159, 160). This may result in potentially geographic, or heritage, specific findings, which may not apply to every location or Hispanic/Latino heritage group. Investments in multi-institutional collaborations across different geographic locations, training, and research capacity building of U.S. academic centers outside of major cities and near emerging Hispanic/Latino communities could fill this gap. Also, collaborations with research teams in Latin America could enhance research capacity building, key information exchange, and best research/clinical practices and to reduce the burden of chronic diseases throughout the Western hemisphere (161–163).

The findings of this analysis need to be interpreted within the context of some limitations. The degree of completeness of grant electronic data restricted our search to RPGs funded in or after 2008 and prevented the coding and analysis of large NIH-led initiatives (14–20, 47–80). RPGs alone do not reflect the NIH's full scope of funding of Hispanic/Latino health research. Although the search engine used RCDC-curated Hispanic/Latino identifiers, 107 applications (out of the 1,237) were not eligible for review because they did not mention Hispanics/Latinos in the planned enrollment tables. Based on NIH funding trends and the methods used in the present analysis, a significant percentage of the projects involved animal models or other non-human studies only. Hence, the overall percentage (4.4%) of all RPGs in which Hispanics/Latinos were truly as a population of interest could have been lower. On the other hand, the approach used to identify eligible RPGs in the present analysis differed from assessing inclusion of women, children and minorities in clinical research, as defined by the NIH OER (164), which considers inclusion as the participation of at least one human research participant from OMB-defined racial/ethnic minority groups and requires monitoring of enrollment of participants by sex and race/ethnicity in clinical research studies (165, 166). Therefore, during the selected time frame, there could have been additional RPGs that planned the inclusion of Hispanics/Latinos but did not identify them as a population of interest. This analysis focused on six health condition areas, and the distribution of funding mechanisms, types of research, age groups, and proposed enrollment might be different for projects in other health condition areas. Finally, we reviewed the proposed research work and planned enrollment of newly funded RPGs, not their completed enrollment and study findings. The final sample size and study findings derived from the RPGs included in this analysis might reveal scientific lessons learned about Hispanic/Latino health that could be evaluated in future analyses.

Twenty-seven years ago, the National Hispanic-Latino Initiative (156), led by U.S. Surgeon General Antonia Novello, identified “the need to increase research in Hispanic health and the participation of Hispanics in research” as top priorities. In 1995, the Secretary of the U.S. Department of Health and Human Services formed the Departmental Working Group on Hispanic Issues (157). This working group recommended plans of action on specific priority areas, including the assurance of “appropriate representation of Hispanics in research and identifying gaps in knowledge of health problems disproportionately affecting Hispanics and taking action to address them” (157). Since then, the NIH has sponsored initiatives—funded through cooperative agreements or contracts—in which Hispanics/Latinos have been the focus or a population of interest (14–20, 47–80). The Hispanic Community Health Study/Study of Latinos (HCHS/SOL), the most recent NIH initiative dedicated to Hispanic/Latino health, has set a foundation to better understand similarities and differences in the prevalence of selected chronic diseases and their risk factors and health events in a cohort of Hispanics/Latinos living in four U.S. cities (14–20).

Although such initiatives have advanced our understanding of the health of Hispanics/Latinos, our analysis posits both the need and the opportunities for continued research in Hispanic/Latino health. The present analysis highlights a percentage of investigator-initiated projects with interest in Hispanic/Latino health for which there is evident room for expansion. The role and scientific contributions of large initiatives and smaller-scale investigator-initiated studies is an ongoing discussion (167–170), which brings to mind a quote attributed to activist Cesar Chavez: “*The fight is never about grapes or lettuce. It is always about the people.”* Considering the diversity of the Hispanic/Latino populations and the complexity and persistence of the health disparities experienced across heritage groups, research on Hispanic/Latino health should propel scientific discoveries that harness cutting-edge science with the imminent health and health-care needs of the populations. While there is no formula for the right or acceptable number of Hispanic/Latino health RPGs that should be funded, a larger proportion of investigator-initiated projects that embrace new paradigms and transform knowledge into action would be an enriching ingredient to the “right mix” (169). It is research that improves health outcomes and changes the portrait of health of the Hispanic/Latino people.

## Data Availability Statement

The datasets for this article are not publicly available because they represent U.S. government funded biomedical research projects and considered confidential. Some of the data presented in our analysis are available through https://report.nih.gov. Requests to access the NIH datasets beyond those available in RePORT should be made to http://www.nih.gov/icd/od/foia/.

## Author Contributions

MA-S led the team. MA-S, LH, TL, SA, LA, LC, PD-N, HN, and AR conceptualized the design of the coding protocol, including use of the internal search engine, and data extraction for review. MA-S, LH, TL, SA, LA, LC, JC, PD-N, HN, and AR participated in some or all the data review (coding). LH and SC did the statistical analyses. MA-S wrote the manuscript. LH, TL, SC, SA, LA, LC, JC, HN, and AR provided critical review and revisions. All authors approved the final version.

## Conflict of Interest

The authors declare that the research was conducted in the absence of any commercial or financial relationships that could be construed as a potential conflict of interest.
